# Cardiovascular Complications of Pregnancy

**DOI:** 10.3390/ijms161023905

**Published:** 2015-10-09

**Authors:** Maria Carolina Gongora, Nanette K. Wenger

**Affiliations:** Department of Medicine, Division of Cardiology, Emory University School of Medicine, Atlanta, GA 30322, USA; E-Mail: nwenger@emory.edu

**Keywords:** pregnancy complications, cardiovascular risk factors, hypertensive disorders of pregnancy, preeclampsia, gestational diabetes

## Abstract

Pregnancy causes significant metabolic and hemodynamic changes in a woman’s physiology to allow for fetal growth. The inability to adapt to these changes might result in the development of hypertensive disorders of pregnancy (hypertension, preeclampsia or eclampsia), gestational diabetes and preterm birth. Contrary to previous beliefs these complications are not limited to the pregnancy period and may leave permanent vascular and metabolic damage. There is in addition, a direct association between these disorders and increased risk of future cardiovascular disease (CVD, including hypertension, ischemic heart disease, heart failure and stroke) and diabetes mellitus. Despite abundant evidence of this association, women who present with these complications of pregnancy do not receive adequate postpartum follow up and counseling regarding their increased risk of future CVD. The postpartum period in these women represents a unique opportunity to intervene with lifestyle modifications designed to reduce the development of premature cardiovascular complications. In some cases it allows early diagnosis and treatment of chronic hypertension or diabetes mellitus. The awareness of this relationship is growing in the medical community, especially among obstetricians and primary care physicians, who play a pivotal role in detecting these complications and assuring appropriate follow up.

## 1. Introduction

Heart disease is the number one cause of death in women older than 65 in the United States across all races and ethnicities. In 2011, one in 3.2 females died of cardiovascular disease totaling 398,035 women who died from heart disease. Almost 40% of women who have a myocardial infarction (MI) die within a year from cardiac complications and almost two thirds who have an MI have no premonitory symptoms of heart disease [1]. Despite the increase in awareness of heart disease mortality for women during the last decade, by 2012 only 56% of U.S. women recognize heart disease as their number one killer [2]. More recently it has become apparent that there are gender and sex differences in the pathophysiology, clinical presentation and response to treatment of CVD. These differences between women and men highlight the need to study sex-specific cardiovascular risk factors, such as hormonal variations and pregnancy-related disorders. Continuous efforts have to be directed toward timely recognition and intervention of CVD in women, and education about signs and symptoms of cardiovascular disease. This manuscript highlights pregnancy-related cardiovascular problems.

For pregnancy related complications, hypertension is the most common, [3] and can occur as gestational hypertension, pre-eclampsia, chronic hypertension, or pre-eclampsia superimposed on chronic hypertension. Women who develop these complications during pregnancy have a greater chance to develop cardiovascular disease later in life [4]. Yet women who develop these complications do not routinely receive long term follow up [5], counseling or risk factor stratification and evaluation. In this review we discuss the metabolic and cardiovascular responses during a normal pregnancy, abnormal responses, specifically hypertensive disorders of pregnancy (HDP), gestational diabetes (GD) and preterm birth. We focus on the current data regarding the association between these pregnancy disorders and future maternal cardiovascular and metabolic risk.

## 2. Hemodynamic Changes during Normal Pregnancy

Major hemodynamic changes begin early in pregnancy. The plasma volume and red cell mass expand as early as the eight weeks, and peak around week 30 [6]. At term, the total plasma volume gain is 1000–1600 mL, corresponding to 30%–50% above baseline. A greater increase in intravascular volume compared to red cell mass results in the dilutional anemia of pregnancy. This is most evident at 30–34 weeks when plasma volume peaks in relation to red cell mass.

An important aspect is that this volume expansion is associated with sodium and water retention. About 1000 meq of sodium and six liters of water are retained and distributed among amniotic fluid, fetus and intra and extracellular spaces [7].

Also, there is an increase and change in distribution of the cardiac output to meet the increased fetal and maternal metabolic demands. Cardiac output increases by 30%–50% above baseline levels during the entire pregnancy, but half of this change occurs by week eight. This significant increase is provoked by, as mentioned above, increases in blood volume, reduction in afterload (decline in systemic vascular resistance) and rise in maternal heart rate [8].

Systemic vascular resistance is reduced due to the high flow, low-resistance circuit in the utero-placental circulation. Vasodilation is thought to be related to decreased sensitivity to norepinephrine and angiotensin, increase in nitric oxide and prostacyclin production and reduction in aortic stiffness. This systemic vasodilation results in an under filling state where plasma renin activity is increased and atrial natriuretic peptide is reduced.

In a normal heart these changes, together with a mild increase in cardiac contractility, assure a significant increase in the total cardiac output without adverse effects on central venous pressure and pulmonary capillary wedge pressure [9].

In normal pregnancy, uterine blood flow increases significantly to allow perfusion of the intervillous spaces of the placenta and support fetal growth. Trophoblasts invade the spiral uterine arteries, vascular smooth muscle cells are lost and replaced by fibrinoid material, turning them into large dilated blood vessels allowing increased perfusion to the placenta [10,11]. Matrix metalloproteinases (MMPs) which degrade the extracellular matrix are upregulated in normal pregnancy [12]. Changes also occur in the aorta: fragmentation of reticular fibers, a decrease in acid mucopolysaccharides, loss of normal corrugation of elastic fibers; and hypertrophy and hyperplasia of smooth muscle cells. MMPs, which degrade the extracellular matrix and connective tissue proteins, decrease in amount and activity in the uterus, placenta and blood vessels leading to increased collagen and growth-restrictive remodeling. These changes contribute to enhanced aortic compliance [13].

The systemic coagulation system is affected as well. Plasma levels of factors VII, VIII, IX, X, XII, Von Willebrand and particularly fibrinogen increase. Protein S levels decrease progressively during pregnancy and the anticoagulant response to activated protein C decreases. Also, during pregnancy blood volume increases, venous stasis occurs as capacitance vessels dilate and venous return decreases due to the pressure from the gravid uterus on the inferior vena cava. All these changes result in a hypercoagulable state with an approximately 20% reduction in prothrombin and partial thromboplastin times [14]. This is why pregnant women are five times more likely than nonpregnant women to develop venous thromboembolism, including deep vein thrombosis and pulmonary embolism [15,16,17].

## 3. Metabolic Changes during Normal Pregnancy

We will discuss the physiologic changes in lipid and glucose metabolism during normal pregnancy.

### 3.1. Glucose Metabolism

During normal pregnancy there is hyperplasia of the pancreatic beta cells resulting in increased insulin secretion and an initial increase in insulin sensitivity followed by progressive insulin resistance. When pancreatic function is not sufficient to overcome the insulin resistance, gestational diabetes develops.

Fasting glucose levels are lower during pregnancy due to increased glycogen storage, peripheral glucose utilization and glucose consumption by the fetus, as well as decreased hepatic glucose production. The relative hypoglycemia allows the mother to preferentially use fat as fuel, and preserve glucose and amino acids for fetal metabolism.

### 3.2. Lipid Metabolism

During pregnancy, complex changes occur in lipid metabolism. Total cholesterol (major high density lipoprotein (HDL) and low density lipoprotein (LDL) particles) and mainly triglyceride levels increase, beginning early in pregnancy and achieving peak levels toward the second trimester [18,19]. Towards the end of pregnancy the maternal consumption of fat increases and storage declines as a consequence of increased lipolytic activity and decreased lipoprotein lipase activity [20]. Maternal hypertriglyceridemia contributes to fetal growth and development and serves as an energy depot for maternal dietary fatty acids [21]. These are caused by estrogen stimulation, hyperphagia and increased lipid synthesis leading to maternal fat accumulation. As mentioned, the main source for maternal energy comes from fat, sparing glucose and amino acids for the fetus.

## 4. Hypertensive Disorders of Pregnancy: Gestational Hypertension and Preeclampsia/Eclampsia

Preeclampsia and eclampsia are important causes of maternal and perinatal morbidity and mortality. These conditions affect 2% to 8% of all pregnancies. Hypertension without proteinuria or symptoms affects approximately 10% of pregnancies. These conditions are of significant importance to the cardiovascular health of women worldwide [22,23].

### 4.1. Diagnosis

Pre-eclampsia is a systemic placenta-mediated disease. It is not a fetal disease; in fact, patients with hydatiform moles can develop preeclampsia.

Chronic hypertension is defined as blood pressure ≥140/90 mmHg diagnosed before pregnancy or before 20 weeks of gestation. Preeclampsia is defined as development of hypertension at more than 20 weeks of gestation associated with either proteinuria or signs of systemic disease. In the absence of proteinuria there should be one of the following: elevated transaminases, thrombocytopenia, visual disturbances, renal insufficiency or pulmonary edema. Preeclampsia can progress to eclampsia, which is characterized by grand mal seizures. Proteinuria during pregnancy is defined as ≥300 mg protein/24 h urine. Urine dipsticks are unreliable and are not used during pregnancy to diagnose proteinuria. Preeclampsia can be classified as early (before 34 weeks) or late (after 34 weeks) of gestation [24].

Complications of preeclampsia and eclampsia include liver rupture, cerebro-vascular accident, pulmonary edema, renal failure and even death [10]. Patients with non-proteinuric preeclampsia but with signs of systemic illness are at higher risk of developing severe hypertension and to deliver prematurely compared to those without systemic illness signs.

### 4.2. Pathophysiology of Preeclampsia

Preeclampsia is believed to result from ischemia of the placenta which in turn releases factors into the maternal circulation that induce the clinical manifestations of the disease.

During normal pregnancy uterine blood flow increases to enable perfusion of the intervillious space of the placenta and to support fetal growth. The physiologic transformation of the spiral arteries of the uterus allow the increase in blood flow, a process in which trophoblasts invade the arterial wall and destroy the media, transforming narrow arteries to dilated vessels enabling adequate perfusion of the placenta. During the first weeks of pregnancy placentation converts the spiral arteries into a low velocity, high-flow compartment. Abnormal trophoblasts or insufficient invasion into the myometrium and the spiral arteries results in shallow placentation and inadequate spiral artery transformation.

Those blood vessels that are not transformed are susceptible to atherosis, fibrinoid necrosis and mononuclear perivascular infiltration. These lesions that resemble atherosclerotic plaque interfere with blood flow to the placenta during the third trimester. Although this abnormality in uterine artery transformation occurs in preeclampsia, it is not unique to this condition and is not sufficient to cause the disease. These abnormal changes have been observed in intrauterine growth retardation, fetal death, placental abruption and preterm rupture of membranes.

Another system involved in the etiology of preeclampsia is the immune system. Natural killer, regulatory T cells and HLA-C molecules modulate maternal immune tolerance to the trophoblasts. In the presence of certain haplotypes these cells can instead induce inadequate placentation, rejection by maternal tissues and induce preeclampsia.

Oxidative stress seems to play an important role in preeclampsia. Increased levels of reactive oxygen species (ROS) might be the result of ischemia-reperfusion injury from deficient conversion of the medial segment of the spiral arteries. Increased ROS exposure leads to protein, lipid and DNA oxidation all of which have been found in placentas from patients with preeclampsia [25].

Other mechanisms involved in preeclampsia include enhanced sensitivity to angiotensin II [26]; disproportionately elevated inflammatory cell activation, specifically granulocytes and monocytes; vasospasm; endothelial dysfunction and platelet consumption [27].

More recently it has been proposed that vitamin D deficiency during pregnancy might be a risk factor for preeclampsia [28,29]. Vitamin D is known to play an important role in vascular inflammation, endothelial dysfunction, vascular stiffness and higher coronary calcium scores, and in some reports, vitamin D supplementation has been shown to reduce inflammation [30,31]. During pregnancy a state of angiogenic abnormalities and increased maternal circulating levels of endothelial adhesion molecules seem to be effects of low levels of vitamin D [32,33]. Whether vitamin D supplementation is beneficial among pregnant patients with low vitamin D levels is unknown and warrants further investigation. Currently there is insufficient evidence to support a recommendation for screening all pregnant women for vitamin D deficiency [34].

### 4.3. Short Term Maternal and Fetal Effects of Preeclampsia and Eclampsia

For women who have mild to moderate hypertension alone, pregnancy outcome is similar to that for women with normal blood pressure. Once proteinuria develops or hypertension becomes more severe outcomes worsen. Preeclampsia-related common complications are poor intrauterine fetal growth and preterm birth, more rarely in the mother eclampsia, stroke, hemolysis, renal failure, low platelets and DIC (disseminated intravascular coagulation). Most maternal deaths are attributable to eclampsia rather than preeclampsia. However, in high income countries where prenatal care is common, fatality case rates have approached less than 1% [35].

Risks for the infants are increased by chronic hypertension, preeclampsia and eclampsia. Maternal-age-adjusted fetal death rates for chronic hypertension and pre-eclampsia were 1.18% and 0.6%, respectively [36]. Increased risk of perinatal mortality in the offspring of mothers with chronic hypertension has also been reported. Premature birth is the leading cause of perinatal morbidity and mortality, and preeclampsia is a common factor in preterm birth. Preeclampsia is an antecedent for up to 12% of infants born small for gestational age and for 20% of those born prematurely [22,37,38].

### 4.4. Long Term Maternal Cardiovascular Risks Associated with Preeclampsia

It was previously thought that the effects of hypertension in pregnancy reversed after delivery and blood pressure values returned to their pre-pregnancy level, *i.e.*, it was seen as a disease of short duration in otherwise healthy young women. However, recent studies have demonstrated that the underlying abnormality, endothelial dysfunction, remains in women who had preeclampsia and that this damage increases the risk of developing cardiovascular disease (CVD) in later life. Even more important, endothelial dysfunction correlates with higher levels of coronary calcium content, which is a predictor of acute coronary events [3,39,40]. Specifically hypertension and preeclampsia during pregnancy was positively associated with the presence of coronary calcium. This association was slightly attenuated with adjustment for body size and blood pressure, but not for serum creatinine [41].

The risk of developing CVD varies among studies. In small studies the risk of developing hypertension after having any kind of hypertensive disorder during pregnancy varied from 1.5 to 20 times. In more recent studies, after changes in the diagnostic criteria for hypertensive disorders of pregnancy and larger numbers of patients, the relative risk varied between 2.3 and 3.7, likely representing more accurate data [42,43,44,45,46]. In a study of women with pregnancy-induced hypertension (HTN) had 11-fold higher age-adjusted risk of developing chronic HTN. In this study, factors that predicted the development of chronic hypertension after having pregnancy induced hypertension included higher Body Mass Index (BMI) and more deliveries before the index pregnancy [47]. Also, HDP are associated with increased risk of venous thromboembolic events and hemorrhagic stroke [48,49].

In terms of metabolic syndrome, 476 women with hypertensive pregnancies and 226 with normotensive pregnancies (controls) were followed for 13 years. After adjusting for age, diabetes mellitus, other features of the metabolic syndrome, smoking and socioeconomic status, women with hypertensive and preeclamptic pregnancies had an increased risk of future hypertension. Long-term hypertension prevalence was 45% in cases and 14% in controls. The association between future development of hypertension was stronger for hypertension than preeclampsia. There was no association between prevalence of Diabetes Mellitus (DM) and hypercholesterolemia and future development of hypertension [50].

In a prospective study of 15,000 women, those with a history of preeclampsia or gestational hypertension had a higher BMI, blood pressure and cholesterol levels. In women with more than one pregnancy with preeclampsia or hypertension these associations were even stronger. Similar findings were reported in a multicenter cohort study in the Netherlands. Three hundred and six women with hypertension during pregnancy and 99 women with normotensive pregnancies were followed for 2.5 years. Blood pressure, BMI and waist circumference were higher in those with hypertension during pregnancy. Biochemical markers such as glucose, glycosylated hemoglobin, cholesterol, triglycerides and C-reactive protein were significantly higher in this group [51]. In another study in the Netherlands women with history of preeclampsia had a two-fold higher prevalence of metabolic syndrome compared to women with history of small for gestational age. The authors suggest that metabolic syndrome prevalence is related to maternal rather than fetal etiology of the placental syndrome [52].

The above-mentioned studies and many others have found that women with hypertensive pregnancy complications have an increased risk for developing cardiovascular disease in later life. It has been postulated that pregnancy may act as cardiovascular stress test to identify women at high risk for cardiovascular disease.

In a study of 4782 women from the Family Blood Pressure study those with a history of hypertension in pregnancy compared to those without such history were diagnosed with hypertension at earlier age, and had higher incidence of coronary artery disease and stroke. These differences remained significant after controlling for race and other risk factors [43]. In a more recent study of more than 90,000 women with history of preeclampsia, these women had more cardiac invasive and non-invasive diagnostic procedures performed (insertion of a stent and a stress test, respectively) and cardiac related hospitalizations (angina and heart failure). There was a linear correlation between the severity of preeclampsia and number of pregnancies with preeclampsia and cardiovascular complications [53].

In a longitudinal follow up study, 300 women with a history of HDP and 94 women with normotensive pregnancies were followed for 2.5 years. Women with history of HDP had significantly higher mean (SD) extrapolated 10-year cardiovascular event and 30-year cardiovascular event risks compared to normotensive women calculated by the Framingham risk scores. The SCORE score and the Reynolds risk score showed similar significant results [54].

In terms of mortality, a study in Jerusalem compared women who died in a 12 year period. Women with history of preeclampsia had approximately 2.1 increased relative risk of death compared to the control group. Death from cardiovascular events accounted for most of the deaths; however no detailed information was available [55]. A retrospective cohort study in Canada that included over a million women showed similar findings. Seven percent of women were diagnosed with either pre-eclampsia, gestational hypertension, placental abruption, or placental infarction. After adjusting for other risk factors, the cardiovascular disease composite outcomes including coronary artery disease, cerebrovascular disease, and peripheral artery disease were two times more frequent in women with a placental syndrome. This risk was further increased by the concomitant presence of intrauterine growth retardation (IUGR) or intrauterine fetal death [56]. In a Taiwan study, 13,000 women with history of hypertension during pregnancy were followed for a median of nine years. Of these women, 46 presented end stage renal disease; compared to women with no history of hypertension during pregnancy, a 10 times higher risk. The risk was higher in women with preeclampsia superimposed on chronic hypertension [57]. In Iceland, 374 women with a history of HDP were compared to the general population. The death rate was higher among women with HDP compared to the control women. Rates were higher for eclampsia than for preeclampsia and hypertension [46]. In a recently published study that examined pregnancy events between 1957 and 1969 factors like hypertension, preeclampsia and preterm birth also were associated to increased CV death, but interestingly glycosuria and hemoglobin decline over the second and third trimester (hazard ration of 4.2 and 1.7, respectively) also predicted CV death [58].

The mechanism by which hypertensive disorders of pregnancy increase the risk of future cardiovascular disease seems to be associated with the persistence of recognized markers of cardiovascular disease after delivery even after resolution of elevated blood pressure. For example, in an observational case-control study women with history of preeclampsia were followed and compared with age-matched controls with uncomplicated pregnancies for at least five years. Ambulatory blood pressure, ultrasound-measured flow-mediated dilatation (FMD), microalbuminuria, carotid intima-media thickness (CIMT) and serum uric acid, as well as clinical and demographic features were measured. Five years after pregnancy, patients who had preeclampsia were more likely to have hypertension and had higher serum uric acid levels, higher microalbuminuria and CIMT levels, and lower FMD values than did the patients who did not have preeclampsia [59]. In a retrospective cohort study in Brazil, data were analyzed from 60 women who delivered at a tertiary care maternity hospital. Thirty women had a history of pregnancy induced hypertension and 30 had no history of complications. Women with a history of hypertension during pregnancy had higher body mass index, systolic blood pressure, LDL levels, and fasting glucose compared with women with no pregnancy complications. Endothelial function as measured by FMD in the brachial artery, was impaired in those with hypertension *vs.* those with normal pregnancies [60]. An important aspect is that severity, parity and recurrence of these hypertensive pregnancy disorders increase the risk of subsequent cardiovascular events [42].

A more recent retrospective study in Ontario focused on the risk of developing arrhythmias and heart failure after developing maternal placental syndrome (MPS, including gestational hypertension, pre-eclampsia and placental abruption/infarction). Over a million (1,130,764) women were included; of those 75,000 (6%) developed a hypertensive disorder during pregnancy. Women with a prior MPS were at significantly higher risk of hospitalization for heart failure or atrial dysrhythmia than women without MPS, starting one year after delivery [61].

## 5. Preterm Birth and Small for Gestational Age—Maternal and Offspring Consequences

It has been proposed that women who have preterm delivery are also at higher risk of developing cardiovascular disease. In fact, offspring birth weight predicts maternal life span. One meta-analysis calculated that, for every standard deviation (roughly 500 g) higher birth weight of the firstborn child, maternal CVD mortality is decreased by 25%. Eight percent of deliveries that are low birth weight (<2500 g) are associated with twice the maternal CVD incidence and mortality of other deliveries. Hypertensive preterm deliveries have a stronger association with maternal CVD outcomes than do normotensive preterm deliveries, although the latter are still associated with a 1.2- to 3-fold increased risk compared with term deliveries [45,62,63,64,65]. A population-based study compared the incidence of cardiovascular morbidity in a cohort of women who delivered preterm (<37 weeks gestation) and those who gave birth at term during the same period. After adjustment for other factors there was a linear association between the number of previous preterm deliveries and future risk for cardiovascular related hospitalizations. This correlation was independent of induced *vs.* natural premature delivery [66]. In a study that included more than 100,000 births, maternal risk of ischemic heart disease or death was associated with delivering a baby in the lowest birth weight quintile for gestational age and preterm delivery [67]. In another study of a cohort of pregnant Finnish women, the relationship between birth dimensions of the offspring, maternal characteristics and mortality was studied. Maternal CVD mortality was inversely related to the birthweight of offspring and women having premature deliveries were also at increased CVD risk [64]. In a similar study in Scotland, maternal mortality from CVD was inversely related to offspring birthweight [68]. These findings were also confirmed by a study that demonstrated a hazard ratio of seven for death from CVD among women who delivered a baby less than 2500 g compared to those delivering babies weighing ≥3500 g [63]. Several studies have reported an association between low birth weight and ischemic heart disease. Studies have shown that women who deliver small for gestational age neonates have a higher incidence of complex cardiovascular events, such as congestive heart failure, hypertensive heart and kidney disease, acute cor pulmonale and even postpartum venous thromboembolism [69]. Also, those with preterm delivery have higher systolic blood pressure and lower HDL values later in life [70,71].

It has also been postulated that the development of hypertensive disorders during pregnancy have a negative effect on the future cardiovascular health of the offspring. Multiple studies have shown a correlation between maternal pregnancy-related hypertension and the development of hypertension in their offspring in childhood and adolescence [72,73,74,75]. Older children born of women with a history of pregnancy-related hypertension also have been shown to have higher levels of blood pressure [76]. When compared with subjects with a normal fetal growth, those born small for gestational age (defined as birth weight < or = −2 SD below the mean) were at increased risk of ischemic heart disease [77]. In an extensive meta-analysis the relative risk for cardiac disease among the offspring of women who developed preeclampsia during pregnancy was 2.33 for the primary outcome of cardiac disease, which included ischemic heart disease, coronary artery disease, myocardial infarction, congestive heart failure, or death from any of the above [73]. A study in New England estimated the association between maternal pregnancy-related hypertension and offspring hypertension later in life. Offspring born to mothers with pregnancy related hypertension had a higher chance to be diagnosed with hypertension and to take antihypertensive drugs later in life [76]. Conflicting data were found in a study where 189 adolescents born to preeclamptic mothers were compared to 300 adolescents born to mothers normotensive during pregnancy. Initially systolic BP seemed higher in those adolescents whose mothers had preeclampsia; however after adjustment for maternal body mass index (BMI) this difference was attenuated. Additional adjustment for adolescent BMI of the offspring further attenuated the difference in SBP. It might be that elevated BP in the offspring of preeclamptic mothers is related mainly to maternal body weight, which in turn is a risk factor for preeclampsia and obesity in the offspring [74].

## 6. Biomarkers Related to Hypertensive Disorders of Pregnancy

Pre-pregnancy body mass index (BMI) is strongly associated with blood pressure in all trimesters and is a strong risk factor for all types of hypertensive disorders of pregnancy. Obese mothers have approximately 4.7 times higher odds ratio of developing gestational hypertension and 2.5 higher odds ratio of preeclampsia than women with normal body weight. Higher pre-pregnancy blood pressure, total cholesterol and LDL levels were associated with the development of gestational diabetes and hypertensive disorders of pregnancy. Also, the more episodes of preeclampsia and the later it occurs in the pregnancy the higher the risk for another episode in future pregnancies [42].

A study that included more than 5000 patients measured different biomarkers including placental growth factor, BMI, mean arterial BP and mean uterine artery resistance to predict development of preeclampsia. This model gave only modest prediction of preeclampsia. Similarly, other studies have not been able to demonstrate a method to effectively predict preeclampsia. Placental growth factor seems able to predict the early onset preeclampsia, which is less common than late onset preeclampsia [78]. Another study performed by the same group used metabolomic technology to try to predict which women would develop preeclampsia. A combination of 14 metabolites measured during the first trimester was predictive of the future development of preeclampsia. These metabolites included amino acids, fatty acids and carnitines [79].

In a study that included 2637 women, 9% developed preeclampsia. Different variables were analyzed to find correlation with the development of preeclampsia. These factors were associated with higher incidence of preeclampsia: chronic hypertension, pre-gestational diabetes, multiple gestation, African American race, prior preeclampsia assisted reproductive techniques and being overweight. Similar associations were found for severe preeclampsia. Being overweight or obese was the most important risk factor for both preeclampsia and severe preeclampsia with an attributable risk percent of 64.9% and 64.4%, respectively [80].

Multiple biomarkers have been identified in the maternal circulation, however no single one has been demonstrated to be clinically applicable. In fetuses with normal chromosome number, low levels of pregnancy-associated plasma protein (PAPP-A) and plasma protein 13 (PP-13) have been associated with the development of preeclampsia, intrauterine growth restriction (IUGR), placental abruption and stillbirth [81]. Increased serum levels of cystatin C in the first trimester are associated with the later development of preeclampsia. Cystatin C inhibits cathepsins, which are expressed in macrophages and trophoblasts and are important in trophoblastic invasion [82]. Inflammatory markers C-reactive protein and PTX3 increase in normal pregnancy as gestation advances, but they have found to be even higher in the first trimester of pregnancies that subsequently develop early-onset preeclampsia [83].

Angiogenic factors like placental growth factor (PlGF) and vascular endothelial growth factor (VEGF) have been found to be reduced in patients who develop preeclampsia [84]. And, since maternal endothelial dysfunction is a feature of preeclampsia, markers of endothelial dysfunction have also been studied. Neutrophil gelatinase-associated lipocalin (NGAL) is a glycoprotein found in neutrophil granules, and was found to be increased in the first trimester of pregnancies later complicated by preeclampsia. Also, P-selectin a cell surface adhesion molecule expressed by endothelial cells and activated platelets, was found to be increased during the first trimester and during preeclampsia [85,86,87].

## 7. Gestational Diabetes

Gestational diabetes mellitus (GDM) affects many women during pregnancy and is enhanced by the epidemic of obesity, increasing age at the time of the first pregnancy, stressful life conditions, sedentary lifestyle with less physical activity and unhealthy nutrition. A diet rich in highly processed foods, high-calories and saturated fat intake has shown to further increase the risk of GDM [88]. The reported prevalence of GDM ranges between 2%–11% [89].

As with hypertensive disorders of pregnancy, the development of gestational diabetes or hyperglycemia during pregnancy represents a risk factor for future maternal type 2 diabetes mellitus, metabolic syndrome, and, with time, cardiovascular disease. GDM, defined as glucose intolerance of varying severity recognized the first time during pregnancy, represents a failure of the pancreas to respond to the progressive insulin resistance of the latter stages of gestation by appropriately increasing beta-cell mass and insulin secretion [90,91,92]; for this reason the American Diabetes Association now advises universal third-trimester screening. After a 75-g or 100-g oral glucose tolerance test women can be classified into normal glucose tolerance, gestational impaired glucose tolerance and GDM. Even milder degrees of glucose intolerance place the mother at increased postpartum cardiovascular risk [93]. GDM is managed initially with diet and lifestyle modification and, if necessary insulin or oral (glyburide or metformin) therapy.

Patients who develop GDM have a higher risk of progression to type 2 DM (T2DM) in the years after the index pregnancy. In some studies it is estimated that GDM and milder gestational hyperglycemia predispose to hyperglycemia soon after delivery, and 20% to 30% of women with GDM will develop T2DM within the first five years postpartum, in part because of persistent pancreatic beta-cell dysfunction [94,95,96]. A meta-analysis that included 28 studies reported an incidence of progression to T2DM ranging between 2.6%–70% at six weeks to 28 years postpartum. This wide range might be explained by different lengths of follow-up, ethnic variation, and the diagnostic criteria used in these studies. Cumulative incidence of T2DM increased markedly in the first five years after delivery and appeared to plateau after 10 years. An elevated fasting glucose level during pregnancy was the risk factor most commonly associated with future risk of type 2 diabetes. It seems that higher glucose levels during pregnancy correlates with higher future risk of type 2 diabetes; it may be possible to stratify risk further based on this variable. Other multiple clinical factors were analyzed but showed variable results among the different studies [97].

Current ADA guidelines recommend glycemic status be reassessed at six to 12 weeks after delivery and every three years thereafter. Women with higher fasting glucose levels during pregnancy and after delivery may warrant more frequent testing and lower-risk women less frequent testing [98].

GDM is also associated with increased risk of metabolic syndrome postpartum [99]. In a study of 487 women, the risk of developing metabolic syndrome increased by up to 10% in those with history of DGM. Women with GDM have a more atherogenic lipid profile by three months postpartum, characterized by increased LDL and apoB and have increased carotid intima-media thickness compared to controls [100,101,102,103,104]. In a prospective study, 917 pregnant women were evaluated and followed for 20 years. After adjustments for BMI and smoking status there was a significant increase in the risk for CVD, hypertension and hospitalization for CVD as gestational HbA1c increased [105]. A study of 2639 women showed that the relationship between GDM and future development of CVD is more robust for women with a BMI > 25 [106]. Markers that can serve as a surrogate of subclinical atherosclerosis even in women without T2DM after having GDM have been proposed. And, a recently published study showed that women with previous GDM have higher carotid intima media thickness, abnormal endothelial dysfunction and increased epicardial fat thickness, indicating their higher risk for cardiovascular disease, beyond their predisposition to future diabetes [99].

Detection of GDM offers clinicians opportunities to care longitudinally for a relatively young population at increased risk for cardiovascular events and to intervene early to modify such risk. The risk of T2DM after GDM is higher in black women and non-European descent women; in these groups impaired glucose metabolism and metabolic syndrome are more prominent, revealing an urgent demand for strategies for cardiometabolic disease prevention for women of these specific ethnicities [107].

## 8. Predictive Models and Risk Reduction

Many groups have tried to formulate models to predict which women will develop preeclampsia. However, despite established clinical characteristics associated with the development of preeclampsia, no single screening test has shown sufficient specificity and sensitivity to be of clinical use.

A multicenter study in New Zealand, Australia, United Kingdom and Ireland attempted to create a predictive model for preeclampsia based on clinical risk factors for nulliparous women and to identify a subgroup at increased risk. Despite finding clinical factors associated with the development of preeclampsia, they concluded that the ability to predict preeclampsia in healthy nulliparous women using clinical phenotype is modest at most. A prior history of preeclampsia is the most consistent predictive factor, which clearly cannot apply to this group—the group with the highest incidence of this condition. These clinical factors include mean arterial blood pressure, body mass index, family history of preeclampsia, family history of coronary heart disease, maternal birth weight and vaginal bleeding. The addition of 20 weeks uterine artery Doppler indices did not improve performance. Surprisingly smoking and alcohol use in the first trimester were associated with lower risk [108,109]. Another study enrolled pregnant women before 15 weeks of gestation [80]. The incidence of preeclampsia was 9%, and the factors associated were chronic hypertension, pre-existing diabetes, multiple gestation, African American race, prior preeclampsia, nulliparity, assisted reproductive techniques, presence of antiphospholipid antibodies, age more than 40, pregnancy interval of ≥10 years and being overweight or obese [80,110]. Chronic hypertension is a risk factor to develop preeclampsia and among low-risk women mean arterial blood pressure (MAP) during the first or second trimester has been proposed to have better predictive value that systolic or diastolic readings alone. The increased MAP in patients who subsequently develop preeclampsia is probably caused by reduced elasticity of the maternal arteries combined with increased vasoconstriction. For high-risk women, the diastolic blood pressure measured between 13 and 20 weeks of gestation was the parameter most predictive for pre-eclampsia [111,112].

The utility of Doppler analysis of the uterine artery in predicting preeclampsia has been extensively studied. The abnormal placentation that characterizes preeclampsia is associated with an increased resistance in the utero-placental circulation. Indicators of the increased resistance are presence of a diastolic “notch” in the Doppler waveform of the uterine artery or an increase in that vessel’s pulsatility index (PI) and the resistive index [109]. A high first trimester PI is however, reversible, and can appear at the end of first trimester in pregnant women with a normal placentation. Therefore, first and early second trimester uterine artery Doppler analysis has relatively low positive predictive value (approximately 21% of preeclampsia cases). In contrast, a normal PI by the end of first trimester is highly predictive for a normal placentation as these women have less than 1% risk of subsequent development of preeclampsia and therefore, a high negative predictive value [113]. A meta-analysis of 18 studies (55,974 women) found an overall sensitivity and specificity of first-trimester uterine artery Doppler in predicting pre-eclampsia of 47.8% (95% CI: 39.0–56.8) and 93.1% (95% CI: 90.6–95.0), respectively. Using this approach the number needed to treat (NNT) with aspirin to prevent one case of early-onset pre-eclampsia fell from 1000 to 173. However, several studies have been published listing reference values for PI by the end of first and second trimester. Based on these publications, it has been concluded that Doppler ultrasound should not be used alone as a first trimester prediction method for preeclampsia but may be valuable as part of other predictive algorithms that also include plasma biomarkers.

In terms of reducing the risk of pre-eclampsia, a meta-analysis that included 18 studies in which different interventions were evaluated, including diet, fatty acid supplementation and life style modifications was performed. It showed that dietary interventions (including fiber, dietary fiber, bran, ispaghula husk, methylcellulose, psyllium, fatty acids, fish oil) reduced risk of preeclampsia by 33%, but no reductions with mixed interventions (diet and physical activity together) or fatty acid supplementation [114].

Therapeutic interventions have also been studied and aspirin has been found to have an effect in preventing preeclampsia. Those women identified as being at risk of preeclampsia have received aspirin, these studies have been compiled and included in meta-analysis. A Cochrane review gathered 46 trials with 32,891 women and found a relative risk reduction of 0.83 (CI 0.77–0.89), NNT of 72 to prevent one case [115]. The PARIS study (Perinatal Antiplatelet Review of International) gathered data from 32,217 women, reported a relative risk of 0.9 (CI 0.84–0.97) and a NNT of 114 [116]. Studies have also found that aspirin offers better prevention if started early during pregnancy, prior to 16 weeks, in agreement with the concept that abnormal placentation plays a role in the genesis of pre-eclampsia [117,118,119].

Also calcium was found to be of benefit in preventing pre-eclampsia. A Cochrane review that included 13 trials and 15,730 women found a relative risk of 0.45 (CI 0.31–0.65) with calcium supplementation compared to placebo; the effect was stronger in women with low baseline calcium intake [120].

## 9. Approaches to Intervention

Until recently the obstetrical clinic was often the first place where women had a measurement of blood pressure, and pre-eclampsia was considered a disease of pregnancy that resolved after delivery. Contemporary data demonstrate that HDP or GDM is a risk factor for development of future CVD. This message should be communicated clearly at discharge from the hospital or at the six-weeks postpartum follow up in the obstetrical clinic. However, most primary care and internal medicine clinicians do not review the obstetrical history as a risk factor. After delivery an important number of these at risk women still do not receive the appropriate screening for hypertension, diabetes, dyslipidemia or CVD. A 2008 study [121]. that utilized the National Health Interview Survey data showed that 93% of women with history of gestational hypertension received the recommended screening for hypertension and 75% received screening for dyslipidemia; these women had higher rates of obesity (43%), CVD (18%), and diabetes mellitus (13%), compared with women without a history of gestational hypertension. In another study a prospective cohort of women were recruited from the postpartum service of a large community-based academic obstetrical hospital after delivery of a pregnancy complicated by gestational diabetes or a hypertensive disorder of pregnancy. At the three month follow up visit, 57% of women with GDM had completed follow-up glucose testing; 97% with hypertension during pregnancy had follow-up blood pressure testing; 57% with either diagnosis recalled ever having completed lipid screening. Women least likely to complete screening tests were those who had no college education, less than a high school level of health literacy, and who were not privately insured [122].

For this reason, recently the American Heart Association 2011 guidelines for the prevention of heart disease in women included hypertensive disorders of pregnancy as a major risk factor for CVD [123,124]. They recommend obstetricians refer the patients to primary care physicians or cardiologists. Few clinics have been created specifically for postpartum CVD counseling. Structured cardiovascular screening programs can ensure adequate follow-up after a hypertensive disorder of pregnancy. It creates a moment to explain and discuss in detail the increased risk of cardiovascular disease and identifies women with comorbidities, such as hypertension, obesity, type 2 diabetes mellitus, and hyperlipidemia. Appropriate referrals of women with evidence of ongoing disease also occur.

Some preliminary reports indicate that women after preeclampsia are willing to participate in such intervention programs and will have improved weight, lipid profile, and vascular function after three months [125,126]. A meta-analysis found that lifestyle interventions after history of preeclampsia decreased cardiovascular risk by 4%–13%. Interventions included tailored aerobic exercise, dietary counseling and restrictions, group meetings, free nicotine patches and telephone support for smoking cessation assistance. This effect is modest, and can be partially explained by relatively short follow periods in the different studies [126]. In another study, the Diabetes Prevention Program trial lifestyle interventions were applied to women with a history of GDM. The multicenter study involved 27 centers including academic and Indian Health Services sites. Women were randomized to standard lifestyle and placebo or metformin therapy or to an intensive lifestyle intervention. Women with history of GDM randomized to placebo had an incidence rate of diabetes 71% higher than that of women without such a history. Among women with a reported history of GDM, both intensive lifestyle and metformin therapy reduced the incidence of diabetes by approximately 50% compared with the placebo group [127].

It has been also proposed that clinics designed for post-partum care, counseling and follow up could potentially reduce the incidence of DM, hypertension and CVD in women with history of hypertensive disorders of pregnancy or GDM. In the General Hospital in Kingston, Canada, The Maternal Health Clinic was created in 2011 to provide postpartum cardiovascular risk counseling or follow-up for women with the pregnancy-related complications, including preeclampsia, GDM, idiopathic preterm birth, placental abruption, and fetal growth restriction. Twenty percent of the target population with an average age of 33 years met criteria of metabolic syndrome and 85% revealed elevated lifetime cardiovascular disease risk. This demonstrates that the creation of programs or clinics designed to follow women with a history of hypertensive disorders of pregnancy have a significant target population to work with. Long term outcome data from groups like this is needed to gain further insight in how we can modify the CVD risk factors after an episode of a HDP [5,128,129]. A small study performed in Canada showed that women with history of preeclampsia who followed up in the Postpartum Preeclampsia Clinic had a significant increase in physical activity and a modest decline in weight. This group is working on a larger sample size and longer duration of follow-up to confirm these findings [130]. A pilot study from a California clinic named Dulce Mothers, from a northern San Diego County federally qualified community health center reported similar findings. This clinic enrolled 84 low-income Latino women with history of GDM; they underwent an eight-week peer-educator led group intervention, with tailoring for Latino culture and recent motherhood. Lifestyle changes and diabetes and cardiovascular risk factors were assessed at study baseline, month three and month six. Participants evidenced statistically significant improvements in lifestyle behaviors, blood pressure and lipids [131].

A group in the Maastricht University Medical Center, the Netherlands proposed a follow up model for patients with history of hypertensive disorders of pregnancy, GDM or preterm birth. This model includes the regular follow up with the obstetrician at six weeks post-partum when BP is measured, followed by a three to six months post-partum visit with either a primary care physician or a cardiologist where BP (preferably including 24-h automated home blood pressure), screening for metabolic abnormalities, body mass index, fasting glucose, lipid profile and dipstick for albuminuria and proteinuria are performed. Patients are referred to a specialist if secondary hypertension is suspected or persistent proteinuria is found. After that a yearly follow up is recommended with the general practitioner with weight and blood pressure screening and every other year glucose and cholesterol measurements. Across these visits, individualized and family centered lifestyle advice for all women should be offered, considering local lifestyle intervention programs. Also, individualized advice is recommended about future pregnancies, and the possible benefits of a healthy lifestyle on recurrence risk. After pregnancy, if pharmacological treatment of high blood pressure and type 2 diabetes mellitus is indicated, treatment of lipid abnormalities should be considered [132].

## 10. Conclusions

Pre-eclampsia occurs in 3% to 5% of all pregnancies, comparable to the prevalence of diabetes mellitus at reproductive age, a well-accepted risk marker for cardiovascular disease. Women with a history of pre-eclampsia have a doubled risk of stroke, cardiac ischemia, or venous thrombosis within 10 to 20 years after pregnancy. They have a four-fold higher risk of hypertension and a three-fold higher risk of type 2 diabetes mellitus.

Growing evidence indicates that women with a history of pregnancy complications, including hypertensive disorders of pregnancy, gestational diabetes, fetal growth restriction and preterm delivery are at increased risk for cardiovascular disease later in life. Findings thus far do not demonstrate a causal relationship between maternal placental disorders in pregnancy and future cardiovascular disease. A more likely explanation relates to a woman’s abnormal metabolic environment that precedes her pregnancy and continues after delivery. This chronic state of abnormal metabolism might create an unfavorable environment during the development of the placental spiral arteries, which can adversely affect fetal health, while negatively affecting the large arteries of a woman’s heart, brain, and extremities over a longer period of time. The current hypothesis is that the development of these pregnancy-related complications indicates an inability to adequately adapt to the physiologic stress of pregnancy and thus reveals the presence of underlying cardiovascular susceptibility to CVD.

Pregnancy offers a unique opportunity during which women at risk of future CVD may be identified. Since pregnancy is one of the few occasions when most young women access the healthcare system on a regular basis, clinicians have an opportunity to implement primary prevention strategies, including health monitoring, lifestyle modifications, and other interventions, that will help reduce the burden of CVD. Studies have shown that women are motivated to change their lifestyle habits during pregnancy or the postpartum period, to optimize fetal, neonatal, and maternal outcomes. In 2011, both the American Heart Association and the European Society of Cardiology included history of pre-eclampsia and in the case of the American Heart Association also GDM as part of CVD risk assessment that would trigger closer monitoring and control of CVD risk factors in women [123,133]. Evidence-based guidelines for screening and interventions remain an unmet need.

Predicting markers for pregnancy complications like pre-eclampsia, gestational diabetes, preterm birth and fetal growth restriction is actively being studied. As a consequence, the next decade is likely to see a paradigm shift in early pregnancy screening, which will require substantial changes to current models of antenatal care.
